# Urban building carbon emissions based on BIM-LCA-GIS

**DOI:** 10.3389/fpubh.2026.1713246

**Published:** 2026-04-16

**Authors:** Zhen-Shuo Zhang, Yang Ding, Kai-Jie Cao, Zhen-Zhen Guo, Shuang-Xi Zhou, Zhi-Peng Lu, Jiang-Liang Dong

**Affiliations:** 1 North Alabama International College of Engineering and Technology, Guizhou University, Guiyang, China; 2 Department of Civil Engineering, Hangzhou City University, Hangzhou, China; 3 Department of Civil Engineering, Jiangxi University of Applied Science, Nanchang, China; 4 School of Civil and Engineering Management, Guangzhou Maritime University, Guangzhou, China; 5 School of Public Administration, Zhejiang Gongshang University, Hangzhou, China; 6 School of Civil Engineering and Architecture, East China Jiaotong University, Nanchang, China

**Keywords:** BIM, carbon emissions, GIS, LCA, prefabricated buildings, urban governance

## Abstract

With the continuous advancement of China’s urbanization process and building technologies, traditional cast-in-situ construction methods have become increasingly incompatible with the concepts of green and healthy development due to significant energy consumption and carbon emissions generated throughout their entire lifecycle, including material production, construction processes, and related upstream and downstream industrial activities, while the advantages of the prefabricated building model have become increasingly prominent in this context; to address the need for in-depth carbon emission analysis of prefabricated buildings, this paper develops an integrated carbon emission analysis model integrating Building Information Modeling (BIM), Life Cycle Assessment (LCA), and Geographic Information Systems (GIS), applies this model to conduct in-depth analysis of carbon emissions at each stage of the prefabricated building’s lifecycle to acquire accurate and specific carbon emission data, and adopts three analytical methods, input-output analysis, and ecological footprint analysis—to perform spatial analysis on the collected data, specifically combining BIM models with the LCA framework to quantify and assess the impacts of different building materials and structural components on energy efficiency and the surrounding environment and using the three computational methods for spatial carbon emission analysis; this integrated model effectively achieves accurate quantification and in-depth analysis of carbon emissions at each lifecycle stage of prefabricated buildings, obtaining specific and reliable carbon emission data and clarifying the spatial distribution characteristics and influencing factors of carbon emissions through spatial analysis; the research findings thus provide valuable insights and practical references for future initiatives to promote low carbon and energy efficient building practices and advance the development of low-carbon buildings, while the established integrated analysis model overcomes the limitations of single-method analysis and improves the accuracy and comprehensiveness of carbon emission analysis for prefabricated.

## 1 Introduction

China’s rapid urbanization and industrial development have led to a higher demand for resilience enhancement, including bridges, buildings, tunnels, etc. (1–5). The urbanization rate in China surged from 37.7% in 2001 to 59.58% in 2018, growing steadily at an average annual rate of 1.21%. The rapid pace of urbanization has driven the healthy and sustainable development of China’s construction industry (6). The construction industry is currently a key technological foundation industry driving the coordinated and stable development of various sectors in China. However, due to the reliance on traditional construction methods and economic management systems in the Chinese construction industry, efficiency is low, and the sector’s overall energy consumption is high. This results in significant waste of natural resources and severe ecological damage due to secondary pollution, and it has prevented the development of a large-scale industrial economy (7). With the rapid advancement of urbanization in China, the fast increase in total construction investment has raised higher demands for the protection of domestic land, resources, that is, wind energy, underground space and the ecological environment, leading to an overall increase in energy production and consumption and a sharp rise in domestic carbon dioxide emissions (8–11). The traditional construction management methods in China can no longer fully adapt to the rapid and healthy development of the modern construction industry (12). It is necessary to reasonably plan and utilize existing building resources, promote the rapid development of traditional prefabricated modern buildings, and achieve a swift transformation of China’s traditional modern construction industry.

The development of prefabricated buildings is not only a key strategic move for the rapid transformation of China’s construction industry but also an important means of improving modern building technology (13, 14). This production technology has significantly improved the operational management efficiency of construction project production lines, enhanced the quality and safety of construction work, shortened the material preparation cycle for building construction, and reduced waste and other energy consumption during construction (15, 16). With the continuous improvement of the prefabricated component construction process in China, a series of national economic support policies, technical support policies, and other related industry standards and measures have achieved notable success in the rapid development of the prefabricated building industry (17, 18). However, challenges still exist in promoting prefabricated construction. For instance, it has primarily developed in major provincial capitals and large cities such as Shanghai, Shenyang, and Hefei, while most other urban areas still lack sufficient market policy promotion and support for prefabricated structures. There is also a lack of political policy support for the relevant manufacturing enterprises, and the quality management systems and market guiding policies for prefabricated building components are still immature (19, 20). Therefore, it is necessary to clarify the specific development path for incentive policies within the complex system of economic and social market policies, conduct long-term systematic quantitative analysis to evaluate the effectiveness of policy implementation, and based on the current market economic resource allocation, provide flexible investment incentive policy recommendations tailored to local conditions. These efforts will inject innovative vitality into the continued development and transformation of China’s prefabricated building materials market.

The construction industry encompasses a wide range of fields and products, making it difficult to accurately determine its carbon emissions in a concentrated manner (21–23). Based on the latest annual statistics from local governments worldwide and international climate change organizations, global carbon emissions from the residential building industry are estimated to be around 5 billion tons per year, which not only includes the total electricity generation and average energy consumption but also the large amounts of carbon emissions potentially generated during use (24, 25). These emissions are also continuously increasing. In recent years, the pace of new urbanization projects in China has been accelerating, which is expected to directly drive a new round of rapid economic growth in the urban construction sector. The challenge lies in effectively reducing carbon emissions from buildings while accelerating urban construction and ensuring the protection of building environment resources (26, 27). Engineers and technical experts in the construction industry have been actively exploring ways to address these challenges. Against this backdrop, this study attempts to explore the relationship between prefabricated buildings and the environment, analyzing the carbon emissions at different stages of prefabricated construction. The goal is to visualize the spatial distribution of carbon emissions in prefabricated buildings, ultimately identifying the potential for energy-saving and emission-reduction in the development of prefabricated buildings.

## 2 Methods

### 2.1 Life cycle environmental assessment (LCA)

Life Cycle Environmental Assessment (LCA) is a method used to evaluate the overall environmental benefits of a product throughout its entire life cycle (28). It is widely adopted in the construction industry and has been extensively applied to engineering projects, building materials, and related equipment. The LCA method comprises four components: Purpose and Scope, Inventory Analysis, Carbon Emission Calculation Model, and Impact Assessment.

Purpose and Scope (29): Based on the theory of life cycle quality assessment in construction engineering, this study compares the carbon emissions at different stages of the entire life cycle of prefabricated composite buildings. It analyzes the key influencing factors of carbon emissions at each stage, including various emission sources and factors that affect the building’s life cycle. Prefabricated buildings involve a wide variety of components with different sizes; for the sake of convenience, a unit size of 1 m^2^ is selected as the functional unit for evaluation. The system boundary covers the entire life cycle, with special emphasis on the manufacturing of building materials, material transportation, on-site assembly, building operation, deconstruction and transportation, maintenance, and material disposal.

Inventory Analysis (30): The inventory analysis stage is the most time-consuming part in calculating the carbon emissions of buildings throughout their life cycle. This stage is based on data collection to assess the main carbon emission components during the entire life cycle, serving as the key foundation for life cycle assessment. The analysis focuses on the resources, energy consumption, and carbon emissions throughout the entire building life cycle. The inventory data includes the types and quantities of resources, energy, and pollutants required by the system during its entire process.

Carbon Emission Calculation Model (31): Carbon emissions mostly come from atmospheric CO_2_, which is often associated with methane (CH_4_) and nitrogen oxides (NO). The International Energy Agency (IEA) has conducted a quantitative analysis of CO_2_ equivalents (CO_2_-eq) in greenhouse gas emissions. The carbon emission probability model for the life cycle of prefabricated energy-efficient buildings refers to the total probability of various greenhouse gases being emitted during each construction phase, and their conversion into CO_2_ equivalents.


$$Q=Q_{c}+Q_{s}+Q_{M}$$ (1)

where, *Q* represents the total carbon emissions throughout the life cycle of prefabricated buildings; *Q_c_*, *Q_S_*, and *Q_M_* refer to the carbon emissions during the construction, use, and disposal phases, respectively.

Impact Assessment (32): Impact assessment involves applying the results obtained from inventory analysis to evaluate the effects of energy consumption and carbon emissions at each stage, while analyzing the contribution and sensitivity of each phase. This assessment enables a deeper understanding of how energy use and carbon emissions during the construction process affect the environment. Key areas are identified in the design phase to address critical issues and reduce the environmental impact of prefabricated buildings on the surrounding areas.

### 2.2 Building information modeling (BIM)

Building Information Modeling (BIM) is not only an intensive application of modern information and digital technologies in the construction industry and other related fields, but also a tool that enables enterprises to make full use of digital information models for construction project management (33). As a technical management tool capable of analyzing large-scale building design data throughout the entire life cycle of a building, BIM data analysis tools can provide construction professionals engaged in life cycle design and analysis with technical support on data and design analysis platforms.

BIM-related software is capable of automatically importing and exporting the material data required for Life Cycle Assessment (LCA), including specific material inventories and detailed construction procedures. To further enhance material intelligence and process automation, BIM can also be integrated with LCA data analysis software, thereby achieving more efficient and effective outcomes.

(1) Integration of BIM and LCA Concepts (34): BIM is not confined to a single life phase of a building but extends across its entire life cycle. It must be capable of integrating data from all stages of the building’s life cycle, which is fully consistent with the design philosophy of LCA.
(2) BIM’s Comprehensive Information and Parametric Management Capabilities (18): BIM inherently features three key characteristics: data visualization, parametric management, and related data integration. Thus, it can integrate all relevant data associated with building components, such as geometric drawings, structural types, material properties, physical manufacturing attributes, and overall design formats. This integration facilitates large-scale data analysis for LCA, offering great convenience.
(3) BIM’s Information Output and Feedback (35): the ultimate goal of LCA is to process the entire life cycle accounting process—including internal environmental impacts—and optimize these processes to minimize environmental effects. BIM not only provides a theoretical foundation for data analysis by managing the overall flow of information but also directly feeds back the results of LCA data analysis into the BIM model. This allows for the direct optimization of large-scale industrial buildings.

### 2.3 Geographic information system (GIS)

A Geographic Information System (GIS) is mainly a technological framework that employs modern digital computing hardware and software to collect, store, manage, compute, analyze, and control geographic data associated with specific locations (36). These locations can range globally and even extend to the Earth’s internal atmosphere. The system processes a variety of geographic data, such as terrain, hydrology, physical features, and natural environments within a specific geographic area. It serves as an automated geospatial data management system for geographic information, grounded in geographical sciences (37).

The product life cycle is not only associated with the different time points and sequences of production activities but also highly dependent on geographic location. Since the production stages of prefabricated components take place at different geographic locations, the same production phase may occur in various places, resulting in differences in production data. For example, the mass production and assembly of components might be carried out in different regions, and different production processes could be implemented at distinct geographic sites (38). Various materials for a project may be sourced from different prefabricated manufacturers and then transported to the construction site through diverse channels. As a result, the production processes of components for the same project may be distributed across different geographic locations.

The production processes of components are closely linked to their geographic environments (39). Therefore, the input and output of components may also be related to their geographic locations, and changes in geographic location can directly affect the impact and evaluation of a component’s entire life cycle. By integrating GIS with LCA, it is feasible to connect abstract component data with spatial location data. This enables a more intuitive and visual presentation of relevant content on a component map, facilitating the exploration of relationship patterns between complex spatial data and component attribute data (40).

### 2.4 Proposed model: LCA + BIM + GIS

Based on the building life cycle quality assessment theory for the entire building life cycle, the primary phases of industrial prefabricated building decoration materials and construction can be divided into six stages: industrial production of building materials and raw material extraction, factory production of building material raw materials, logistics and transportation of building material raw materials, on-site assembly and installation of building material raw materials, use and maintenance of building materials, and demolition and resource recovery management. In accordance with the LCA engineering standards proposed by the International Cooperation Development Organization in September 2005 for the internationalization of industrial building materials and project quality standards, the key task is to construct a carbon emission input inventory for various building structures throughout their life cycles. The main work involves compiling various basic building statistics related to life cycle quality for each phase, as these data serve as the key basis for life cycle measurement and evaluation calculations. The primary data inputs and outputs of the building carbon emission inventory include raw materials, energy, and other available resources used in buildings, while the outputs mainly consist of the main structure and body of various types of buildings, as well as pollutants from construction machinery (41). During the evaluation and calculation phase, special attention is usually paid to the actual comprehensive utilization rates of different building energies, as well as the operational efficiency and utilization rates of various construction machinery.

Carbon emissions during the building construction phase can be divided into three main stages (42): (1) on-site production of prefabricated components, (2) transportation and delivery of prefabricated components, and (3) on-site handling and assembly. The on-site production of prefabricated components, as well as their transportation and installation, can be analyzed and quantified based on the basic elements of labor, machinery, and materials. Therefore, the basic equations for calculating safety and carbon emissions during the construction of prefabricated structures are defined based on these three factors—workers, equipment, and materials.

The carbon emissions from construction workers are calculated by multiplying the total carbon emission factor for the labor in each sub-unit project by the corresponding unit quantity for that project. The total carbon emission factor for each sub-unit is determined by the carbon emissions of the labor in each specific process of the sub-unit and the corresponding work quantity for each process, that is, (43).


$$E_{\mathrm{hum}}=\sum_{i=1}^{n}Q_{n}C_{\mathrm{hum},n}$$ (2)

$$C_{\mathrm{hum},n}=\sum_{j=1}^{m}Q_{m}C_{\mathrm{hum},m}$$ (3)

where, *E_hum_* is carbon emissions from construction workers during the building construction period; *C_hum,n_* is carbon emission factor for labor in the sub-unit project during the construction period; *C_hum,m_* is carbon emission factor for labor in a specific unit process during the building construction period; *Q_n_* is quantity of work for sub-unit project *n*; *Q_m_* is quantity of work required for unit process *m* to complete the sub-unit project; *m* is number of processes in the sub-unit project; *n* is number of sub-units in the project; *I* represents the *i*-th sub-unit; *j* represents the *j*-th process.

The carbon emissions from raw materials in a project are calculated by multiplying the total carbon emission factor for raw materials in each sub-unit by the corresponding unit quantity for that sub-unit. The total carbon emission factor for raw materials in each sub-unit is determined by the carbon emissions of raw materials in each specific process of the sub-unit and the corresponding work quantity for each process (33).


$$E_{\mathrm{mot}}=\sum_{i=1}^{n}Q_{n}C_{\mathrm{mot},n}$$ (4)

$$C_{\mathrm{mot},n}=\sum_{j=1}^{m}Q_{m}C_{\mathrm{mot},m}$$ (5)

where, *E_mot_* is carbon emissions from raw materials during the building construction period; *C_mot,n_* is carbon emission factor for raw materials in the sub-unit project during the construction period; *C_mot,m_* is carbon emission factor for raw materials in a specific unit process during the building construction period.

The carbon emissions from various equipment are calculated by multiplying the total carbon emission factor for equipment in each sub-design project by the corresponding unit quantity for that equipment. The total carbon emission factor for equipment in each sub-design project is determined by the carbon emissions of the equipment in each specific process of the sub-design and the corresponding work quantity for each process (44).


$$E_{\text{mech}}=\sum_{i=1}^{n}Q_{n}C_{\text{mech},n}$$ (6)

$$C_{\text{mech},n}=\sum_{j=1}^{m}Q_{m}C_{\text{mech},m}$$ (7)

where, *E_mech_* is carbon emissions from equipment during the building construction period; *C_mech,n_* is carbon emission factor for equipment in the sub-design project during the construction period; *C_mech,m_* is carbon emission factor for equipment in a specific unit process during the building construction period.

**Figure 1:**
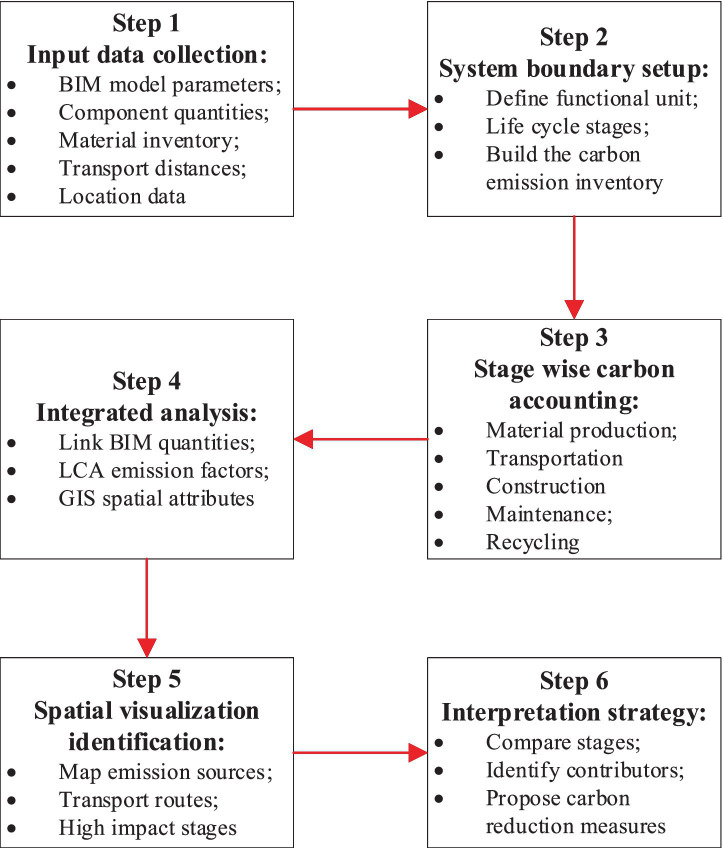
Flowchart of proposed model.

Based on the above equations, the carbon emissions generated during the on-site construction phase mainly include equipment carbon emissions, labor carbon emissions, and raw material carbon emissions and the flowchart of the model proposed in this article is shown in Figure 1.


$$E_{\text{construction}}=E_{\mathrm{mot}}+E_{\mathrm{hum}}+E_{\text{mech}}+E_{\mathrm{mat}}$$ (8)

where, *E_construction_* is carbon emissions generated during the on-site construction phase of the building; *E_mat_* is carbon emissions from the materialization phase.

## 3 Case analysis

### 3.1 Carbon emissions in the material production stage

Based on the model established in previous research, determining each related parameter remains crucial for the model calculation. The parameters include carbon emission factors and other related life activity measurement data. These activity measurement data can either be obtained from actual measurements or industry statistical data. For LCA, evaluation can be done from two basic aspects: longitudinal and horizontal. The longitudinal comparison focuses on each life cycle phase, while the horizontal comparison is based on carbon emission factors for components, modules, and structures in modern industrialized buildings. According to this design approach, prefabricated components like inner core wall panels, composite floor panels, staircases, balconies, air conditioning panels, and hanging panels are constructed and prefabricated simultaneously as needed, as shown in Tables 1, 2.

**Table 1:** Quantity statistics of prefabricated parts in the project.

Number	Prefabricated component model	Type	Quantity	Weight/ton	Production method
1	Prefabricated exterior wall	72	426	1,318	Fixed flat mold
2	Prefabricated interior wall	40	275	815	/
3	Prefabricated composite panels	28	541	425	/
4	Prefabricated balcony and hanging board	29	175	124	Mold table + edge touch + magnetic box
5	Prefabricated air conditioning panel	3	82	24	/
6	Prefabricated staircase	72	47	36	Forming and modeling

**Table 2:** Project prefabricated material cost statistics.

Building material	Unit	Quantity	Amount (10,000 RMB)	Proportion	Cumulative proportion
Steel	t	234.96	84.59	35.37%	32.86%
Concrete	m^3^	1,333.99	40.02	16.73%	54.66%
Doors and windows	m^2^	1,770.79	35.42	14.81%	68.1 1%
Block	Thousand piece	60.19	36.11	15.10%	80.06%
Wood	m^3^	56.36	8.45	3.53%	89.31%
Thermal insulation material	t	57.24	2.29	1.91%	93.75%
Other	/	/	30.05	12.54%	100%

This paper is primarily based on the energy emission factors provided by the IPCC for China and, in combination with the development status of domestic clean energy, summarizes and proposes a table of carbon emission factors for China’s energy sector that is adapted to the current energy situation in the country, as shown in Table 3.

**Table 3:** Energy and carbon emission factors required in the project.

Energy type density	Density (t/m^3^)	Emission factor (kgCO_2_/kg)	Energy type density	Density (t/m^3^)	Emission factor (kgCO_2_/kg)
Standard coal		2.772	Fuel oil	0.95	3.241
Raw coal	0.5–0.75	1.980	Gasoline	0.74	2.988
Coke	0.5	3.046	Diesel oil	0.86	3.164
Crude oil	0.81	3.069	Kerosene	0.8	3.101
Liquefied petroleum gas	0.717×10^−3^	3.17	Refinery gas	0.7×10^−3^	3.011
Oilfield natural gas	0.7×10^−3^	2.162	Electric	/	0.723 kg/kWh

When performing the calculation and correction of parameters for different regions, we use a comprehensive calculation method as follows: For the primary batch production, transportation, and plastic product processing of various building materials, a comprehensive heat accounting for the carbon emissions of non-CO_2_ gases due to radiation between regions is used. The impact of non-CO_2_ greenhouse gases can be considered in the comprehensive heat accounting process. We prioritize using a calculation method for equivalent electricity price calorific value to calculate the correlation of the heat emission data related to non-CO_2_ gases. A comprehensive accounting is made for the recycling rate of several major building materials in different regions. As mentioned earlier, the recycling rate for materials like ceramic tiles and concrete is generally set at 55%, the non-CO_2_ gas recycling rate for metals is 75%, the recycling rate for plastics is 10%, and demolition waste is also considered as a non-effective gas recycling rate, as shown in Table 4.

**Table 4:** Recycling coefficient of major building materials.

Material	Recovery rate	Material	Recovery rate
Bricks, Tiles, Ceramics, Concrete	0.55	Wood	0.2
Glass	0.7	Technology	0.75
Plastic	0.1	Tar and asphaltene products	0.75
Mixed demolition waste	0	/	/

### 3.2 Carbon emissions in the construction stage

The carbon emissions in construction can primarily be divided into two main stages: project transportation and construction. The transportation of building materials for the project is mainly carried out using three modes of transportation: rail, shipping, and highways. For this construction project, the transportation of prefabricated components predominantly uses highways. The vehicle types and highway transport capacity requirements are detailed in Table 5 of the project.

**Table 5:** Pre-finished transport model configuration.

Vehicle type	Vehicle model	Installed construction type	Vehicle number	Installed construction quantity (block)
I-type	Normal 12.5	Laminated board	562	15
	Semi trailer load capacity			
	31 ton transport vehicle			
II-type	Normal 12.5	Air conditioning panel	81	6
		Balcony board	92	
	Semi trailer load capacity	Balcony hanging board	82	
	31 ton transport vehicle	Stairs	46	
		Interior wall panel	271	28
III-type	Low board 12.5	Exterior wall panel	426	41
	Semi trailer load capacity			
	31 ton transport vehicle			
Total			1560	90

The carbon emissions generated during material transportation are proportional to the weight of the transported materials, the transportation distance, and the carbon emission factor of the transportation.


$$C_{21}=\sum_{i=1}^{n}\sum_{j=1}^{m}m_{\mathrm{ij}}\times p_{j}\times L_{j}$$ (9)

where, C_21_ is carbon emissions during the transportation of building materials (kg), *n* is type of building material, *m* is type of transportation method, *I* is the *i*-th type of building material, *j* is the *j*-th type of transportation method, *M_ij_* is quantity of the *i*-th building material transported by the *j*-th transportation method, *P_j_* is carbon emission factor per unit mass per unit distance for the *j*-th transportation method [kg CO_2_/(km.unit)], *L_j_* is transportation distance of the *i*-th building material by the *j*-th transportation method (km).

From Tables 6, 7, we can clearly observe that the transportation of building materials using prefabricated components is divided into three types of common diesel vehicle models. The estimated total number of trips for transporting the prefabricated components is 1,579. Additionally, some site-cast materials, which substitute for building raw materials, require long-term continuous transportation during their delivery process. As it is difficult to comprehensively calculate the average transport distance for substituted building materials due to their frequent relocation and long-term transportation, we can refer to the average long-distance transport statistics from Taiwan for this purpose. This data can be used to estimate the average transportation distance for the substituted building materials’ long-term delivery. Super-large diesel road transport vehicles are generally divided into two types: diesel-powered vehicles for general-purpose use and those for specific diesel road transport. The common diesel transport vehicles in Tables 3–5 are all diesel-powered road transport vehicles. Prefabricated components are comprehensively calculated according to the tonnage density relationship of prefabricated parts and reinforced concrete in the building. The average transportation distance for the design of building goods should be 65.38 km, with an average vehicle load of 31 tons. The total number of transport trips is 1,579, and the average total weight density of the building’s transportation process is 48,949 tons. Consequently, the average carbon emissions for the transportation of a single building’s prefabricated components in the construction process is 52,884.72 kg.

**Table 6:** Average transport distance of building materials.

Building material	Average transportation distance/km	Building material	Average transportation distance/km	Building material	Average transportation distance/km
Sand	28.99	Cement products	65.38	Precast concrete	65.38
Steel	122.72	Cement	65.57	Furniture (non-metallic)	51.37
Plywood	86.16	Glass	98.84	Mineral building materials	57.75
Aluminum doors and windows	128.08	Coating	103.81	Wood	59.01
Bricks and wall materials	57.75	Ceramics	106.38		

**Table 7:** Carbon emission factor of different transportation methods.

Type of shipping	CO_2_ emission factor kgCO_2_/(10^4^t. km)	Type of shipping	CO_2_ emission factor kgCO_2_/(10^4^t. km)
Highway transportation (gasoline)	2,004	Highway transportation (diesel)	1,983
Railway transportation	91.3	Waterway transportation	183
Transport aviation	10,907	/	/

### 3.3 Determination of carbon emissions parameters for prefabrication construction

During the on-site construction of the building’s main structure, the primary materials used for main casting include hydrogen, oxygen, acetylene, diesel, gasoline, water, and electricity. The main on-site construction process involves preparing for the casting of the building’s main structure, including the installation and connection of structural components and joints. These activities must be carried out simultaneously to ensure the building’s safety, structural integrity, and stability. The installation and connection of the frame structure with the main building require casting preparation, while the connection of the building wall panels and the installation of floor slabs also necessitate on-site casting preparation.

Since the internal structural frame and high-rise floors of the building still require cast-in-situ construction, there may be substantial consumption of construction raw materials. In addition, during the construction process, internal and external maintenance, decoration, and other consumption processes may occur, resulting in the extensive use of auxiliary building materials for interior and exterior decoration. The specific consumption quantities are detailed in Table 8.

**Table 8:** Construction of the construction of the construction.

Building material	Unit	Quantity	Transportation distance/km	Carbon emissions (kgCO^2^/10^4^t.km)	Carbon emission calculation/kg
Steel	t	205.67	122.72	1,983	5,005.06
Concrete	m^3^	1,352.66	65.38		43,842.60
Mortar	m^3^	1,126.744	65.57		36,626.31
Coating	kg	29,143.97	103.81		599.94
Cement	t	437.53	65.57		5,689.00
Paint	t	4.05	103.81		83.37
Water	t	4,101	0		0
Total	/	/	/		91,846.23

Therefore, by applying the carbon emission impact factors and the dispersed consumption factors for non-original materials related to transportation, the average carbon emissions during the non-original material consumption phase of the construction preparation stage for the project’s complete vehicle assembly line are estimated to be 1,835,358.34 kg. Similarly, the carbon emissions for the transport of raw materials during the construction preparation stage are estimated to be 91,846.23 kg. Through a comprehensive analysis of the carbon emissions generated during the consumption, transportation, and usage of construction materials at each stage of the building assembly process, it can be preliminarily determined that the total average carbon emissions per unit of material used during the building material phase of construction is 4,046,931.06 kg. When converted, this corresponds to 544.14 kg/m^2^ of carbon emissions per unit of floor area for the final construction. Therefore, the total unit carbon emissions calculated for the construction material usage process during the assembly stage reflect a higher efficiency in material design, as all carbon emissions from the various raw materials used in the construction process are evenly distributed across the stages of building material usage.

### 3.4 Comparative analysis of carbon emissions with traditional construction

In the design case used in this study, the prefabrication rate for individual prefabricated components in prefabricated construction is generally 17%. This is distinct from using a certain percentage of prefabricated components in traditional concrete prefabricated construction. The volume of prefabricated concrete components is approximately a certain proportion of the total volume of prefabricated structural concrete components. The case study in this article primarily assumes that the prefabricated area ratio used in prefabricated construction design refers to the contribution rate of the prefabricated construction area to the total building area calculation. Therefore, the case study research assumes that the total CO_2_ emissions per square meter for the design of traditional prefabricated construction components are as follows.


$${\mathrm{EM}}_{co_{2}}=\frac{E_{{\mathrm{co}}_{2}}}{S\times V}$$ (10)

where, ${\mathrm{EM}}_{{\mathrm{co}}_{2}}$ represents the CO_2_ emissions per square meter of prefabricated components, $E_{{\mathrm{co}}_{2}}$ represents the total CO_2_ emissions, *S* is the total building area, and V represents the prefabrication rate of the prefabricated construction.

For the total greenhouse gas emissions from the traditional cast-in-place building structure, the emission calculation method involves selecting a large high-rise residential building as a case study. Specifically, the building in the case is used to analyze the total greenhouse gas emissions. The total construction area for building in this event is determined to be 13,858.78 square meters, with 31 floors. It features a reinforced concrete shear wall or steel frame structure. The main materials and building usage of the cast-in-place structure for each floor are shown in Table 9.

**Table 9:** Traditional current pouring.

Material	Material usage
Steel bar	788.327 t
Concrete	6,855.704m^3^
Coal stone hollow brick	519,750 piece
Concrete bricks	473.92m^3^
Cement	938.359 t
Lime	151.41 t

**Figure 2:**
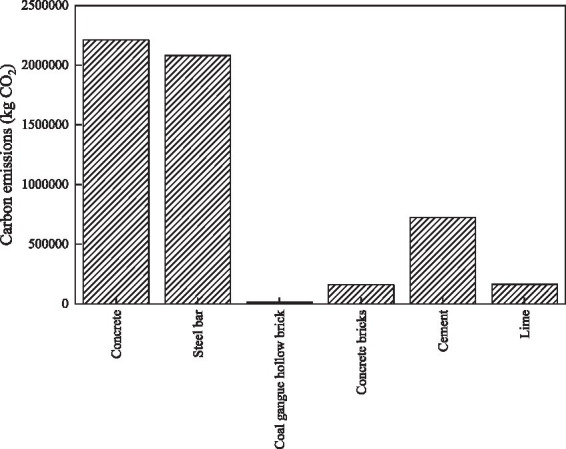
Traditional casting construction of carbon emissions emissions.

The specific greenhouse gas emissions from each building material are shown in Figure 2. From the structural diagram, it is clear that, similar to traditional prefabricated cement buildings, the greenhouse gas emissions during the cement production phase of concrete hollow core reinforced cement still dominate the market, accounting for 41.31 and 38.89%, respectively. The next significant emission source is cast-in-place cement, which emits 723,000 kg of CO_2_. One of the main reasons for these emissions is the need to use artificial cement and lime in cast-in-place concrete buildings, which contributes significantly to greenhouse gas emissions. Although the use of coal-mined hollow bricks is noticeably higher than that of artificial concrete hollow bricks, the greenhouse gas emissions during the production process are notably lower for coal-mined hollow bricks. This demonstrates that the cement production for concrete hollow bricks contributes more significantly to the greenhouse gas emissions during the production process.

### 3.5 Spatial distribution of carbon emissions from prefabricated building components

Through GIS modeling, the distribution pattern of the total transport space for the total greenhouse harmful gas emissions of prefabricated building components can be directly obtained. The prefabrication plant, various steel and cement production plants, and the pipelines at the building construction site are displayed as transport point sources one by one in the distribution map. Meanwhile, the transportation routes of various material transport plants and the distribution patterns of pipeline sources are also presented in the map sequentially. From the distribution map, it can be clearly observed that the headquarters of the steel and cement production plant is the farthest from this prefabrication plant in terms of transportation distance. Although its total greenhouse harmful gas emissions per kilometer are not the highest, the long transportation route results in its total greenhouse harmful gas emissions being the highest among all railway transports, reaching 16,048.03 kg.

Other prefabricated raw material factories are relatively more concentrated. Some large-scale prefabricated steel bar material production plants are closer in distance on the route map, and the road network in these areas is relatively dense. Therefore, from these route maps, it can often be clearly found that within a certain route area covered by the map, the concentration of greenhouse gas emissions from prefabricated steel bar material plants and other steel bar raw material factories is relatively high. Based on the analysis model of the spatial distribution of greenhouse gases and carbon emissions in prefabricated cast components, it can be concluded that the location selection of prefabricated component production plants has a significant impact on the greenhouse gas and carbon emissions of industrial prefabricated components. Since the location of the prefabrication plant directly determines and affects the transportation distance of components from production to construction sites, to further explore the impact of the location selection of industrial prefabricated component plants on the production and transportation processes of components, as well as the spatial distribution of greenhouse gases and carbon emissions from prefabricated components, this paper selects four major large-scale industrial prefabricated cast component manufacturers in Shenzhen and its surrounding areas for on-site investigation and statistical analysis. This paper assumes that each prefabricated cast component factory in the research scope provides prefabricated components for the on-site construction of the same project, and the production and transportation processes of prefabricated component manufacturers, as well as the sources of various material factories, are from the same location.

It can be observed that when prefabricated component manufacturing plants are established in Shenzhen and its surrounding areas, the economic input required to reduce carbon emissions during transportation increases with the distance between the plant and the construction site. An analysis of the raw material procurement and component delivery processes shows that transporting prefabricated components to construction sites contributes the most significantly to greenhouse gas and carbon emissions due to the large volume of the transported components. Since each delivery vehicle has a limited capacity, multiple trips are required; thus, minimizing the transportation distance between prefabricated component factories and construction sites becomes crucial for reducing emissions. In addition, high-demand materials such as steel reinforcement and lightweight wall composite concrete require the proximity between production facilities and construction sites. For example, locating modern prefabricated component factories entirely within Shenzhen can significantly reduce carbon emissions from vehicle exhaust during transportation.

However, it is important to note that prefabricated component factories are often located in remote mountainous areas rather than urban centers, mainly for two reasons: (1) Land cost: Prefabricated component plants require a large amount of land resources, and the high land prices in urban areas encourage enterprises to locate factories away from cities to reduce costs. (2) Environmental impact: These factories generate significant pollution, so being close to urban areas would be harmful to residential and commercial districts. Furthermore, China’s prefabricated component transportation infrastructure is still underdeveloped compared with international standards. The logistical advantages of prefabricated concrete structures over traditional cast-in-place concrete structures are limited due to two factors: Transportation challenges: Large prefabricated components require specialized vehicles and face constraints in route optimization. Industry immaturity: The small number of domestic prefabricated component factories restricts opportunities for transportation optimization. In contrast, traditional cast-in-place concrete structures rely on flexible supply chains, where materials are transported directly from factories to scattered construction sites—a more resource-efficient arrangement under current conditions.

## 4 Emission reduction strategies

### 4.1 Carbon emission reduction measures during the construction phase

Adopt Progress Control Methods: Establish a balanced relationship between the actual carbon emissions and the construction progress of the project. By applying relevant calculation principles, the monthly carbon emission target for the project can be derived. At the end of each month, the actual construction progress is compared with the theoretical carbon emission target to assess the deviation. Statistical analysis is conducted using the theoretical carbon emissions of completed projects, the actual design carbon emissions, and the deviation between the theoretical carbon emissions of uncompleted projects, so as to analyze the construction progress and carbon emission deviation.

Optimize Technical Improvement Measures: Improve efficiency and update evaluation methods by optimizing design schemes. The evaluation of the optimized design scheme should focus on three key aspects: the assessment of the overall construction design scheme, the management of the overall construction process, and the evaluation procedures. The evaluation criteria should include the overall design and management plans, while the evaluation process should mainly consider construction technology, economy, and application effects—especially the correlation with total carbon emissions. The evaluation process includes initial screening, analysis of key attribute indicators, comprehensive analytical analysis, and comprehensive evaluation, followed by optimal selection.

Clarify Management Guidance Measures: Set clear management goals, define responsibilities, and strengthen quality supervision. Defining responsibilities involves formulating project management rules, appointing project leaders, and assigning specific responsibilities to sub-project leaders, who are also granted actual authority in quality assessment. It also includes quality control, covering key indicators such as project quality, investment, progress, safety, and carbon emissions. The two management aspects refer to contract management and project information management. Finally, strengthening quality supervision includes overseeing various on-site carbon emission indicators, including carbon emission monitoring, statistics, auditing, public disclosure, and the establishment of corresponding management systems.

### 4.2 Measures for reducing carbon emissions during the construction materials production phase

Use of New Materials and Dry Processing Technology (45–48): By using new materials and dry processing technology for cement production, energy utilization efficiency can be significantly improved, offering considerable potential for carbon emissions reduction. Currently, the average energy efficiency in the cement production industry has a 20–40% potential for energy savings. Therefore, companies need to continually upgrade their cement production equipment and optimize manufacturing processes to effectively reduce industrial energy consumption. This approach could result in a 20–40% reduction in CO_2_ emissions.

Improving Raw Material with AI Utilization (49–51): Reducing the use of large quantities of limestone, chemical raw materials, and nuclear fuel, and continuously updating and replacing them with large industrial waste materials, can significantly reduce overall industrial CO_2_ emissions. For instance, although China currently has 1 billion tons of raw materials that could be replaced with industrial waste, the overall utilization rate is low, around 30%. Therefore, efforts should be made to increase the utilization rate, which would substantially lower overall carbon emissions.

### 4.3 Measures for reducing carbon emissions during the construction logistics and transportation phase

Optimization of Logistics Transportation Plans: In the design process of international logistics for secondary transportation, a variety of transportation factors need to be comprehensively considered. These factors include the duration of secondary logistics operations, types of transportation modes, the combination of transportation routes, and the selection of component loading and transportation schemes. Only through the analysis and integration of all these factors can the international logistics for secondary transportation be organized more effectively and reasonably. For primary international logistics, unnecessary secondary logistics should be minimized as much as possible.

Use of Clean Energy Vehicles: Clean energy vehicles, commonly referred to as new energy vehicles, are those that use environmentally friendly fuels such as clean gasoline or natural gas instead of traditional fuels. These vehicles feature lower energy consumption and fewer pollutant emissions, providing an effective guarantee for the carbon emission reduction process during the first phase of industrial logistics vehicle transportation. For instance, logistics vehicles using clean fuels such as natural gas, liquefied gas, and LPG make significant contributions to reducing carbon emissions.

## 5 Conclusion

Against the backdrop of the construction industry’s low-carbon transformation, this study constructs a BIM-LCA-GIS integrated model to address the data fragmentation and lack of spatial perspective in prefabricated building carbon emission assessment, and verifies the model’s effectiveness through an empirical study on a practical project. The key conclusions are drawn as follows:

(1) The BIM-LCA-GIS integrated model realizes the efficient fusion of engineering parametric data, life cycle emission data and geographic spatial data. It forms a standardized technical framework of “data acquisition-quantitative accounting-spatial analysis,” which effectively improves the accuracy of carbon emission inventory statistics and fills the gap of spatial traceability in traditional prefabricated building carbon emission analysis.
(2) For the case project with a 17% prefabrication rate, the material production stage dominates the carbon emissions during the construction period, followed by the transportation stage. The low prefabrication rate and long transportation distance are the main reasons why the unit area carbon emission of the case project is higher than that of traditional cast-in-place buildings of the same type.
(3) Sensitivity analysis confirms that the prefabrication rate is a key factor affecting carbon emissions: every 10% increase in the prefabrication rate can reduce the unit area carbon emission by 8–10%. GIS spatial analysis further shows that shortening the transportation distance between component factories and construction sites is an effective way to cut down transportation carbon emissions.
(4) The targeted emission reduction strategies proposed for the material production, on-site construction and logistics transportation stages have strong operability, which can provide direct technical reference for construction enterprises and relevant management departments to carry out low-carbon management of prefabricated buildings.

This study is limited to the carbon emission analysis of the material production and construction stages of low-prefabrication-rate residential projects, and does not cover the operation and demolition stages. Future research will expand the LCA system boundary, incorporate more types of projects and dynamic influencing factors to further improve the universality and prediction ability of the model.

## Data Availability

The original contributions presented in the study are included in the article/supplementary material, further inquiries can be directed to the corresponding author.
